# A Child with a c.6923_6928dup (p.Arg2308_Met2309dup) *SPTAN1* Mutation Associated with a Severe Early Infantile Epileptic Encephalopathy

**DOI:** 10.3390/ijms19071976

**Published:** 2018-07-06

**Authors:** Valentina Rapaccini, Susanna Esposito, Francesco Strinati, Mariella Allegretti, Elisabetta Manfroi, Francesco Miconi, Mariabernarda Pitzianti, Paolo Prontera, Nicola Principi, Augusto Pasini

**Affiliations:** 1 Department of Systems Medicine, Unit of Child Neurology and Psychiatry, “Tor Vergata” University of Rome, 00133 Rome, Italy; rapaccinivalentina@gmail.com (V.R.); b.pitzianti@libero.it (M.P.); augusto.pasini@uslumbria2.it (A.P.); 2 Unità Sanitaria Locale (USL) Umbria 2, Viale VIII Marzo, 05100 Terni, Italy; fstrinati@libero.it; 3 Paediatric Clinic, Department of Surgical and Biomedical Sciences, Università degli Studi di Perugia, Piazza Menghini 1, 06132 Perugia, Italy; francesco.miconi90@gmail.com; 4 Department of Child Neurology and Psychiatry, 47122 Forlì, Italy; mariella.allegretti@uslumbria2.it; 5 Department of Genetics, Santa Maria Hospital, 05100 Terni, Italy; e.manfroi@aospterni.it; 6 Medical Genetics Unit, S. Maria della Misericordia Hospital, 06132 Perugia, Italy; paolo.prontera@ospedale.perugia.it; 7 Università degli Studi di Milano, 20122 Milan, Italy; nicola.principi@unimi.it

**Keywords:** early infantile epileptic encephalopathy, nervous system damage, *SPTAN1*

## Abstract

Early infantile epileptic encephalopathies (EIEEs) are a group of neurological disorders characterized by early-onset refractory seizures, severe electroencephalographic abnormalities, and developmental delay or intellectual disability. Recently, genetic studies have indicated that a significant portion of previously cryptogenic EIEEs are single-gene disorders. *SPTAN1* is among the genes whose mutations are associated with EIEE development (OMIM# 613477). Here, a case of the c.6923_6928dup (p.Arg2308_Met2309dup) *SPTAN1* mutation associated with a severe EIEE is reported. This case shows that mutations in the α20 repeat in the C-terminal of αII spectrin can be associated with EIEE. Duplication seems essential to cause EIEE. This causation is not demonstrated for amino acid deletions in the same spectrin residues. Reportedly, children with p.(Asp2303_Leu2305del) and p.(Gln2304_Gly2306del) deletions have childhood-onset epilepsy and no or marginal magnetic resonance imaging abnormalities, suggesting that not only the location but also the type of mutation plays a role in conditioning nervous system damage. Further studies are needed for a better understanding of the phenotype/genotype correlation in *SPTAN1*-related encephalopathies.

## 1. Introduction

Early infantile epileptic encephalopathies (EIEEs) are a group of neurological disorders characterized by early-onset refractory seizures, severe electroencephalographic abnormalities, and developmental delay or intellectual disability [1]. Seizures starting in the first year of life including the neonatal period might have a favorable course, such as in infants presenting with benign familial neonatal epilepsy, febrile seizures simplex or acute symptomatic seizures. However, in some cases, the onset of seizures at birth or in the first months of life have a dramatic evolution with severe cerebral impairment [2,3]. This group of disorders includes early infantile epileptic encephalopathy, also known as Ohtahara syndrome, early myoclonic encephalopathy, epilepsy of infancy with migrating focal seizures, infantile spasms syndrome (also known as West syndrome), severe myoclonic epilepsy in infancy (also known as Dravet syndrome), and myoclonic encephalopathies in nonprogressive disorder [3]. Structural brain abnormalities, inborn metabolic defects and acquired brain damages are the major underlying causes. However, for years, the pathogenesis remained unknown in a number of EIEE cases. Recently, genetic studies have indicated that a significant portion of previously cryptogenic EIEEs are single-gene disorders [4]. 

*SPTAN1* is among the genes whose mutations are associated with EIEE development (OMIM# 613477) [5]. This gene encodes αII spectrin, one of the flexible submembranous scaffolding proteins involved in the stabilisation of cell membranes. Two α- and five β spectrin subunits have been identified. These subunits are assembled in an antiparallel side-by-side manner into heterodimers that can form end-to-end tetramers that integrate into the membrane cytoskeleton. As heterotetramer integrity is essential for neuronal process development and inhibitory synapse formation [6], mutation of *SPTAN1* can lead to significant neurological disorders [7]. However, clinical manifestations of 34 individuals with *SPTAN1*-associated disorders do not always resemble those described as EIEE [8]. Mild cases have been reported, suggesting that different *SPTAN1* variants can be associated with different clinical manifestations. Attempts to establish genotype/phenotype correlations have suggested that mutation site and type can play a role in conditioning development and severity of nervous system damage. However, due to the low number of patients with *SPTAN1*-related disorders and the relatively high number of gene mutations described thus far, phenotype/genotype correlations remain undefined at present. Here, a case of the c.6923_6928dup (p.Arg2308_Met2309dup) *SPTAN1* variant associated with a very severe neurologic disease is reported. As only two cases with this mutation have been described to date, this case report can contribute to an understanding the role of different *SPTAN1* mutations in the determination of neurological damage. 

## 2. Discussion

Recent advances in genetics have determined that a number of epilepsy syndromes that occur in the first year of life are associated with genetic etiologies [9]. An early genetic diagnosis can save time and overall cost by reducing the amount of time and resources expended to reach a diagnosis. Furthermore, a genetic diagnosis can provide accurate prognostic information and, in certain cases, enable targeted therapy. As genetic information accumulates, genetic testing will likely play an increasingly important role in diagnosing pediatric epilepsy. Most cases of *SPTAN1* mutations are associated with EIEE. Syrbe et al. analysed characteristics of 20 patients with pathogenic or likely pathogenic *SPTAN1* mutations and compared them with those of subjects with the same genetic abnormality in previously published studies [8]. These authors reported that 62% of affected children presented with EIEE. These patients typically presented with early onset of recurrent, intractable seizures associated with severe developmental delay. Several patients had West syndrome with typical electroencephalographic findings that tended to evolve into disorganized slow background activity with frequent multifocal spikes. Delayed and incomplete myelination associated with brain atrophy was the main finding from MRI and can be considered a hallmark of *SPTAN1*-associated encephalopathy. Death in the first years of life is common. However, approximately 30% of the cases presented a mild phenotype with seizures starting after infancy, relatively good response to antiepileptic drugs and no risk of early death. 

Interestingly, many of the mild cases had *SPTAN1* mutations located far from the C-terminal region. In contrast, most of the severe cases had in-frame deletion/duplication mutations located in the last two αII spectrin repeats in the C-terminal region. As these repeats are required for α/β spectrin heterodimer association, altered heterodimer formation between the spectrins is thought to cause instability and aggregation of spectrin scaffolds [10]. In-vitro studies conducted in both transfected primary neurons and patient-derived lymphoblastoid cells with mutations in the last two αII spectrin repeats have shown that the αII/βII spectrin heterodimers containing mutant spectrin were more unstable than those with normal spectrin. Moreover, instability was associated with aggregate formation [8]. This leads to loss of integrity of the axon initial segment (AIS). The AIS is essential for normal nervous system development and function, and it is unsurprising that AIS impairment can lead to severe EIEE [11]. 

## 3. Materials and Methods

### 3.1. Case Report

**Figure 1:**
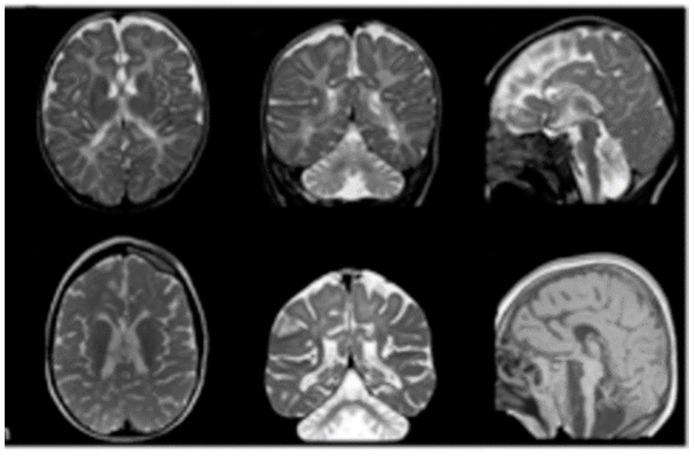
Magnetic resonance imaging. Images are 1.5 to 3 T and include T1-weighted, T2-weighted and fluid-attenuated inversion recovery (FLAIR) sequences. Structural abnormalities include cerebellar and brainstem atrophy, dilated ventricles and subarachnoid spaces, thinning of the corpus callosum and hypomyelination.

The child described here was first seen at the Paediatric Unit of the Santa Maria Hospital, Terni, Italy, when he was 3 years old. The hospital admission was required by parents for further evaluation of an already diagnosed EIEE. The child was born from eutocic delivery after a 39-week regular gestation. Neurological problems emerged during the first days of life when a significant hypertonus of the lower limbs was clearly evident. In the following weeks, repetitive, difficult-to-treat seizures occurred. Moreover, neuromotor and psychic development was very poor. At admission, the clinical manifestations included epileptic encephalopathy with tonic and myoclonic seizures and spasms refractory to polypharmacy, severe cognitive disability, and severe postural spastic paresis with a dystonic-myoclonic component (both cortical and truncal myoclonias at rest and in action). Magnetic resonance imaging (MRI) showed complex brain malformations such as pontocerebellar hypoplasia, corpus callosum atrophy and simplified cortical architecture (Figure 1). These characteristics were also associated with a posterior laryngomalacia. The child underwent tracheotomy and percutaneous gastrostomy because of his low aptitude for swallowing. Congenital cardiac cardiopathies were not observed at the cardiological consultation, and no congenital alterations were seen in renal ultrasound.

**Figure 2:**
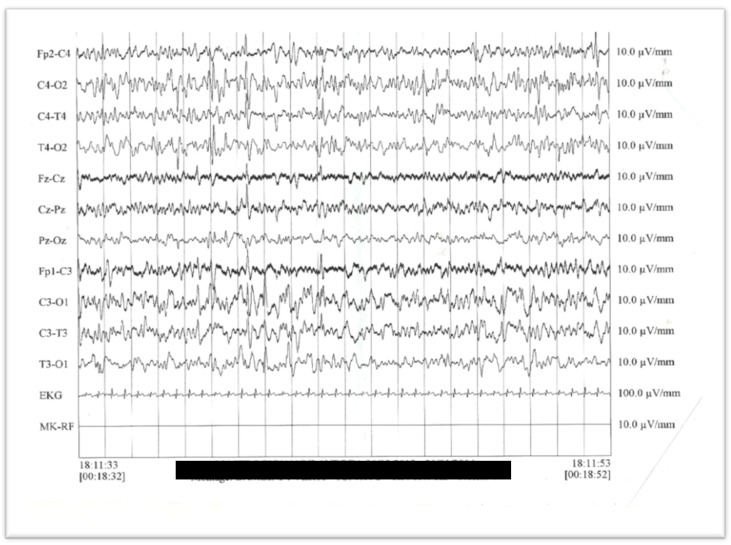
The electroencephalogram (EEG) obtained when the patient was 3 years old reveals diffuse cortical electrical anomalies in the centro-temporal areas of both cerebral hemispheres; theta-delta band rhythms, with intermittent repetitions of sharp waves, are mainly evident on the left hemisphere where they assume a subcontinuous morphology.

The patient has continued to show repeated daily critical episodes, characterized by a polymorphic tonic semeiology with an inconstant clonic component, tonic spasms in extension, tonic deviation of the head on the left and inflection adduction of the right upper limb, and clonus of the head and upper left limb. The electroencephalogram (EEG) showed a poorly organized pattern for the age of the subject. On the centro-temporal derivations of the two hemispheres, theta-delta band rhythms were present, followed by intermittent repetitions of sharp waves, mainly evident on the left hemisphere where they assume a subcontinuous morphology. The cortical electrical anomalies were evident in the centro-temporal areas of both cerebral hemispheres, especially on the left side (Figure 2).

### 3.2. Genetic Analysis

When he was 5 years old, clinical information and blood samples were obtained after approval from the Ethics Committee of Umbria Region and signed written informed consent by both parents. Genetic analysis was carried out using genomic DNA isolated from peripheral blood. A target resequencing method with a GS FLX Titanium 454 Roche platform was used. A heterozygous mutation, c.6923_6928dup, was located in the *SPTAN1* gene. This alteration results in the duplication p.Arg2308_Met2309dup.

**Figure 3:**
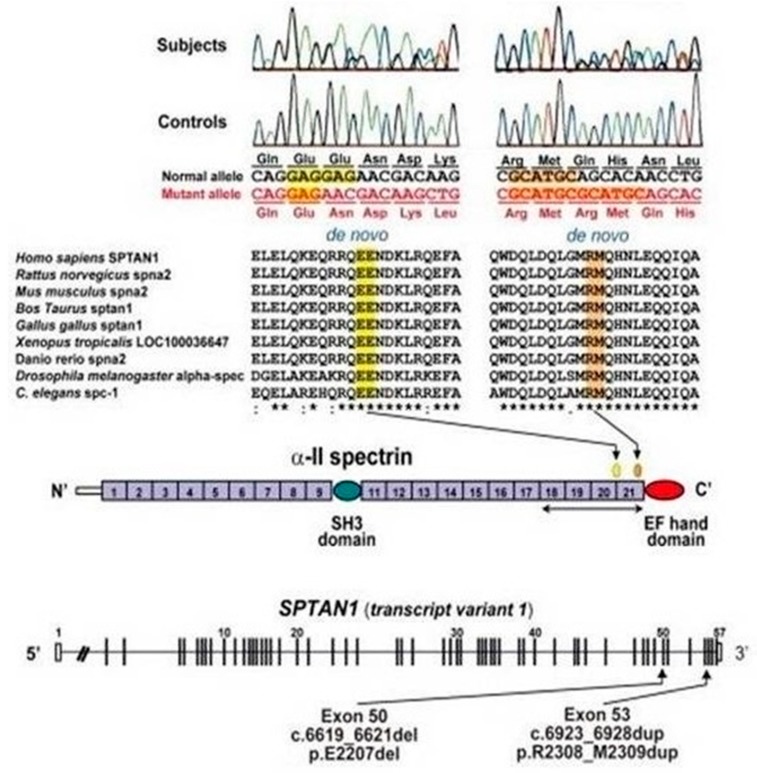
Schematic representation of *SPTAN1* gene (transcript variant 1) consisting of 57 exons. Exon 37 of transcript variant 1 is missing in variant 2. Two distinct de-novo mutations were found at evolutionary conserved amino acids in triple helical repeats (spectrin repeats). α-II spectrin consists of 22 domains, including 20 spectrin repeats, and the mutations occurred within the last four spectrin repeats, which are required for α/β heterodimer association.

The duplication was confirmed by the Sanger sequencing method, and subsequent analysis on parental DNA defined its de-novo origin (Figure 3). 

## 4. Conclusions

The case described here shows that mutations in the α20 repeat in the C-terminal of αII spectrin can be associated with EIEE. The clinical history and MRI findings of this child are quite similar to those of two children previously described with the same *SPTAN1* mutation [12,13]. Moreover, they are similar to those of other patients carrying different mutations in the α19–α20 repeats [8]. However, importantly, all of these cases, including the one described here, have duplication of amino acid residues within the amino acid stretch 2303–2309 in the αII spectrin repeat 20. Duplication seems essential to cause EIEE. This causation is not demonstrated for amino acid deletions in the same spectrin residues. Reportedly, children with p.(Asp2303_Leu2305del) and p.(Gln2304_Gly2306del) deletions have childhood-onset epilepsy and no or marginal MRI abnormalities [8], suggesting that not only the location but also the type of mutation plays a role in conditioning nervous system damage. Further studies are needed for a better understanding of the phenotype/genotype correlation in *SPTAN1*-related encephalopathies.
